# Integrated whole-heart computational workflow for inverse potential mapping and personalized simulations

**DOI:** 10.1186/s12967-016-0902-0

**Published:** 2016-05-25

**Authors:** P. Bhagirath, A. W. M. van der Graaf, J. de Hooge, N. M. S. de Groot, M. J. W. Götte

**Affiliations:** Department of Cardiology, Haga Teaching Hospital, Leyweg 275, 2545 CH The Hague, The Netherlands; Department of Cardiology, Thorax Center, Erasmus Medical Center, ‘s Gravendijkwal 230, 3015 CE Rotterdam, The Netherlands

**Keywords:** Personalized cardiac model, Whole-heart simulation, Whole-heart inverse potential mapping, FEM based cardiac simulations, Patient-specific therapy

## Abstract

**Background:**

Integration of whole-heart activation simulations and inverse potential mapping (IPM) could benefit the guidance and planning of electrophysiological procedures. Routine clinical application requires a fast and adaptable workflow. These requirements limit clinical translation of existing simulation models. This study proposes a comprehensive finite element model (FEM) based whole-heart computational workflow suitable for IPM and simulations.

**Methods:**

Three volunteers and eight patients with premature ventricular contractions underwent body surface potential (BSP) acquisition followed by a cardiac MRI (CMR) scan. The cardiac volumes were segmented from the CMR images using custom written software. The feasibility to integrate tissue-characteristics was assessed by generating meshes with virtual edema and scar. Isochronal activation maps were constructed by identifying the fastest route through the cardiac volume using the Möller–Trumbore and Floyd–Warshall algorithms. IPM’s were reconstructed from the BSP’s.

**Results:**

Whole-heart computational meshes were generated within seconds. The first point of atrial activation on IPM was located near the crista terminalis of the superior vena cave into the right atrium. The IPM demonstrated the ventricular epicardial breakthrough at the attachment of the moderator band with the right ventricular free wall. Simulations of sinus rhythm were successfully performed. The conduction through the virtual edema and scar meshes demonstrated delayed activation or a complete conductional block respectively.

**Conclusion:**

The proposed FEM based whole-heart computational workflow offers an integrated platform for cardiac electrical assessment using simulations and IPM. This workflow can incorporate patient-specific electrical parameters, perform whole-heart cardiac activation simulations and accurately reconstruct cardiac activation sequences from BSP’s.

**Electronic supplementary material:**

The online version of this article (doi:10.1186/s12967-016-0902-0) contains supplementary material, which is available to authorized users.

## Background

Inverse potential mapping (IPM) and simulations of cardiac activation are promising computational techniques for non-invasive assessment of rhythm disorders [1, 2]. Recent studies have examined the role of simulation models for personalizing catheter ablation strategies [3, 4]. Furthermore, catheter ablation guidance by IPM shows a higher accuracy when compared to conventional mapping procedures [5, 6].

In general, IPM requires a (computational) mesh representing the thoracic and cardiac volumes to reconstruct cardiac activation sequences from body surface potentials (BSP). Similarly, realistic simulations demand for a patient-specific mesh, incorporating functional information about tissue characteristics such as electrical conductivity, mechanical deformation and fiber orientation [3, 4, 7]. In contrast to meshes used solely for visualization (shells), the computational meshes for these purposes require topologically correct segmentations.

Although the currently available models are useful, they are very time consuming [4], or too comprehensive (multiple parameters) [7], and therefore not ready for use on a routine basis in the clinical arena.

In addition, none of the currently available methods provide an integrated whole-heart (topologically correct) mesh, incorporating both the atria and ventricles. This limits a comprehensive and integrated study of whole-heart electrical interaction.

This article proposes a comprehensive finite element model based whole-heart computational workflow suitable for IPM and efficient personalized simulations. The clinical feasibility of reconstructing IPM was explored using BSP’s of healthy volunteers and patients with premature ventricular contractions (PVC’s).

Subsequently, the simulation features were explored by generating activation maps (isochrones) in different models of human hearts, both normal and with structural heart disease.

## Methods

**Fig. 1:**
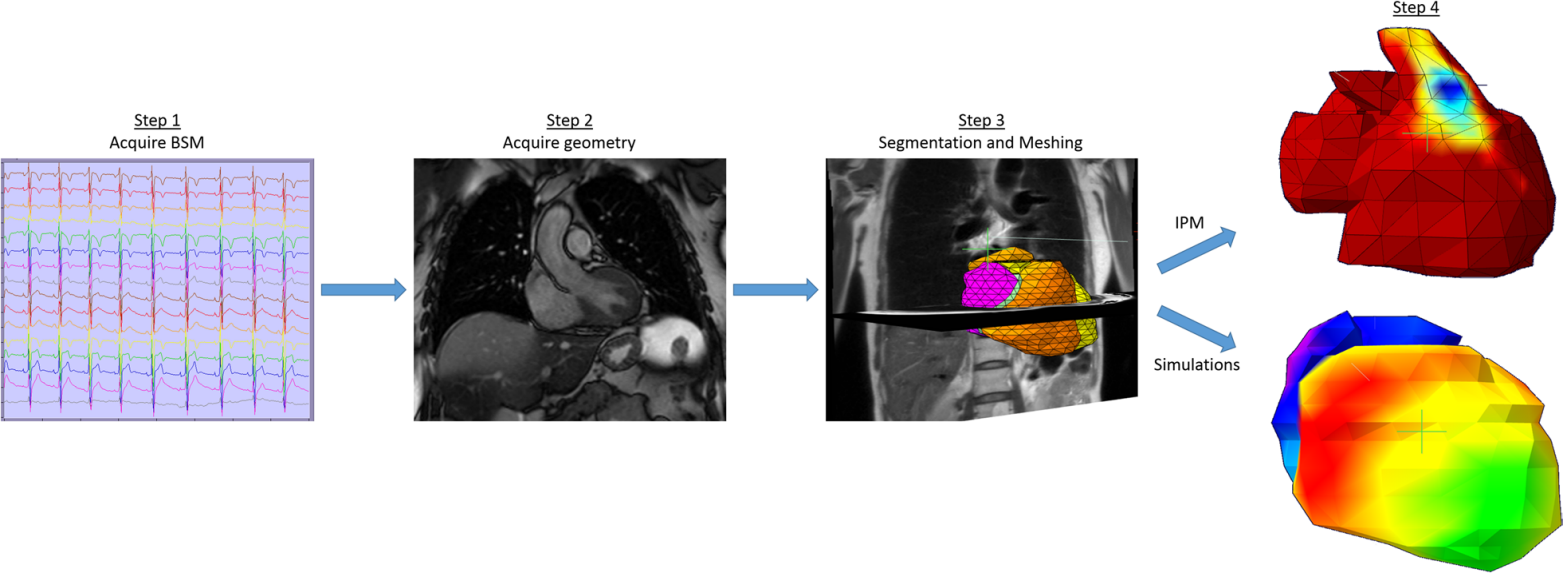
Workflow of the integrated whole-heart computational models. *Step 1* represents the acquisition of the multichannel body surface potentials; *step 2* acquisition of geometry in 3D (1D shown); *step 3* depicts the topologically correct wholeheart segmentation in the transversal and coronal plane of a volunteer; *step 4* the workflow can generate both inverse potential maps (*top*) and simulations (*bottom*) using the information supplied in the previous steps

The computational workflow for whole-heart electrical assessment consists of four steps (Fig. 1). These steps involve (1) acquisition of BSP, (2) acquisition of subject specific geometry using cardiac magnetic resonance imaging (MRI), (3) topologically correct segmentation and generation of the computational mesh and (4) utilizing the mesh for reconstructing cardiac surface potentials or conducting simulations.

### Study population

Three healthy volunteers and eight patients with symptomatic or therapy resistant premature ventricular contractions (PVC) participated in this investigation. The study complied with the declaration of Helsinki and received approval from the local ethical committee (METC Zuidwest Holland study number NL38156.098.11) and the institutional scientific board. Written informed consent was obtained from the volunteers.

### Body surface potential acquisition

An MRI scout scan was performed to approximate the position of the heart with respect to the thorax. Subsequently, 62 (+3 limb) electrodes were applied to the subject’s torso, centralized over the heart. Body surface potentials (BSP) were acquired using a 65 channel ActiveTwo system (BioSemi B.V., Amsterdam, The Netherlands). Once the acquisition was completed, the electrode locations were marked with MRI markers enabling accurate identification of the electrode positions.

### Image acquisition

MRI studies were obtained on a 1.5 Tesla Aera scanner (Siemens Healthcare, Erlangen, Germany). Blackblood imaging was performed using a Half-Fourier Acquisition Single Shot Turbo Spin Echo (HASTE) pulse-sequence to acquire three perpendicular stacks (axial, coronal and sagittal). The scan provides coverage from the neck till lower abdomen.

Images were acquired during free breathing using navigator gating (diaphragm) with 1 mm window. ECG gating was used to acquire views during the diastolic phase of the cardiac cycle. Typical imaging parameters were: a spatial resolution of 1.2 × 1.2 × 6 mm, TR/TE 744/42 ms and flip angle = 160°.

### Whole-heart computational model

#### Anatomical and electrical components

A topologically correct description of the whole-heart anatomy was constructed using the different cardiac structures such as atrial and ventricular endocard and epicard, the inter-ventricular septum (IVS) and inter-atrial septum (IAS), and tricuspid and mitral valvular plane (Additional file 1: Video 1). These different structures were used to generate the cardiac volumes required to represent a whole-heart.

In order to incorporate the electrical pathways and to account for the differences in conductivity, the conduction system of the heart was also modelled. The origin of activation for sinus rhythm was defined at the lateral border of the superior vena cava and right atrium junction, approximating the location of the sinus node. The right and left bundle branches were also defined.

#### Topology

A formal description was composed to define the topological properties of the segmentation result. This description contained a definition of all tissue volumes involved in terms of topological elements, i.e. patches, shells and hulls.

##### Patch

A patch is a set of facets denoting an elementary surface part. Example: patches.heart_LA_outer = Patch (index) denotes the outer surface of the left atrium. A unique color index describes the relation between the topological description and the colored planes that make up the segmentation.

##### Shell

A shell is a set of patches denoting a closed surface. Example: shells.heart_outer = Shell (patches.heart_LA_outer,_patches.heart_RA_outer,_patches.heart_LV_outer,_patches.heart_RV_outer) denote the whole outer surface of the heart.

##### Hull

A hull is a three dimensional piece of tissue having uniform properties which may be irregularly shaped. A hull is bound by one or more shells where the first-mentioned shell is the outer surface of the hull and remaining shells are the inner surfaces of holes in the hull. Example: hulls.heart = Hull (shells.heart_outer, shells.heart_LA_inner, shells.heart_RA_inner, shells.heart_LV_inner, shells.heart_RV_inner) denote the hart walls. In this example the heart is simplistically pictured as a closed volume with four holes, two for the atria and two for the ventricles.

#### Image segmentation and mesh generation

The three perpendicular stacks of MRI images were loaded in a custom developed software tool. Subsequently, the pre-defined anatomical and electrical components were segmented. The extra-cardiac thoracic volumes (lungs and thorax) were assigned conductivity values (Σ) described in the literature; lungs (0.04 S/m) and thorax (0.2 S/m) [8].

To investigate the feasibility of incorporating tissue properties, two segmentations were created containing pre-defined regions of edema (speed 0.2 m/s and Σ 0.0325 S/m) and scar tissue (speed 0 m/s and Σ 0 S/m) respectively.

The software tool was used to generate a script containing the geometrical description of the topologically correct segmentation result. From this script input a high quality 2D/3D mesh can be instantly generated. This mesh is compound, i.e. it is divided in labeled sub-meshes conforming organ boundaries, and suitable for computational use e.g. sub-volume properties and boundary conditions. The script generated by the segmentation tool was used as input to the GMSH mesh generator to construct the computational mesh [9].

### Inverse potential mapping and simulation of cardiac activation

The IPM’s were reconstructed by multiplication of the BSP’s with

$$\left( {{\text{T}}^{\text{T}} {\text{T}} +\uplambda^{2} {\text{I}}} \right)^{ - 1} {\text{T}}^{\text{T}}$$

where T is the transfer matrix and λ = 0.01. As described previously, this provided the cardiac surface potentials from which the IPM was derived [10].

Simulations were performed using a fixed conduction velocity model. In concordance with the literature, an effective conduction velocity of 0.6 m/s was defined for both atrial and ventricular myocardium [11]. Based on the same literature the bundle branches were assigned a speed (2 m/s) and delay (0 m/s).

The simulations were performed (1) computing direct connections between all mesh nodes using the Möller–Trumbore algorithm [12] followed by (2) solving the shortest path problem amongst the computed paths using the Floyd–Warshall algorithm [13]. The mesh nodes were assigned a potential versus time activation curve. A standard potential curve was chosen for this purpose. For each individual node, this curve was offset by the local activation time which was computed by the first come first serve principle. Based upon the results of the simulations an isochronal activation map was generated.

## Results

Baseline characteristics of the three volunteers and eight patients (N = 11) are provided in Table 1. All volunteers had normal electrocardiograms. In both volunteers and patients the MRI examination was performed in 12 ± 2 min; there were no complications. Clinical characteristics are provided in Table 1.

**Table 1:** Clinical characteristics of study patients

	Volunteers	Patients
n	3	8
Age (years)	28 ± 3	46 ± 13
BMI	22.1 ± 1.4	25.2 ± 6.7
Female (%)	1 (33 %)	7 (88 %)
LVEF (%)	55 ± 2	50 ± 3

### Whole-heart computational model

For each individual, five different volumes were defined at the atrial level consisting of the left and right atrium, IAS and the mitral and tricuspid valvular planes. The ventricular volumes were defined as the left and right ventricle and IVS (Additional file 1: Video 1). Limited interaction (<5 min) was required to create the meshes with (virtual) structural heart disease.

Mesh generation was typically completed in seconds and required no further post-processing.

### IPM in sinus rhythm

**Fig. 2:**
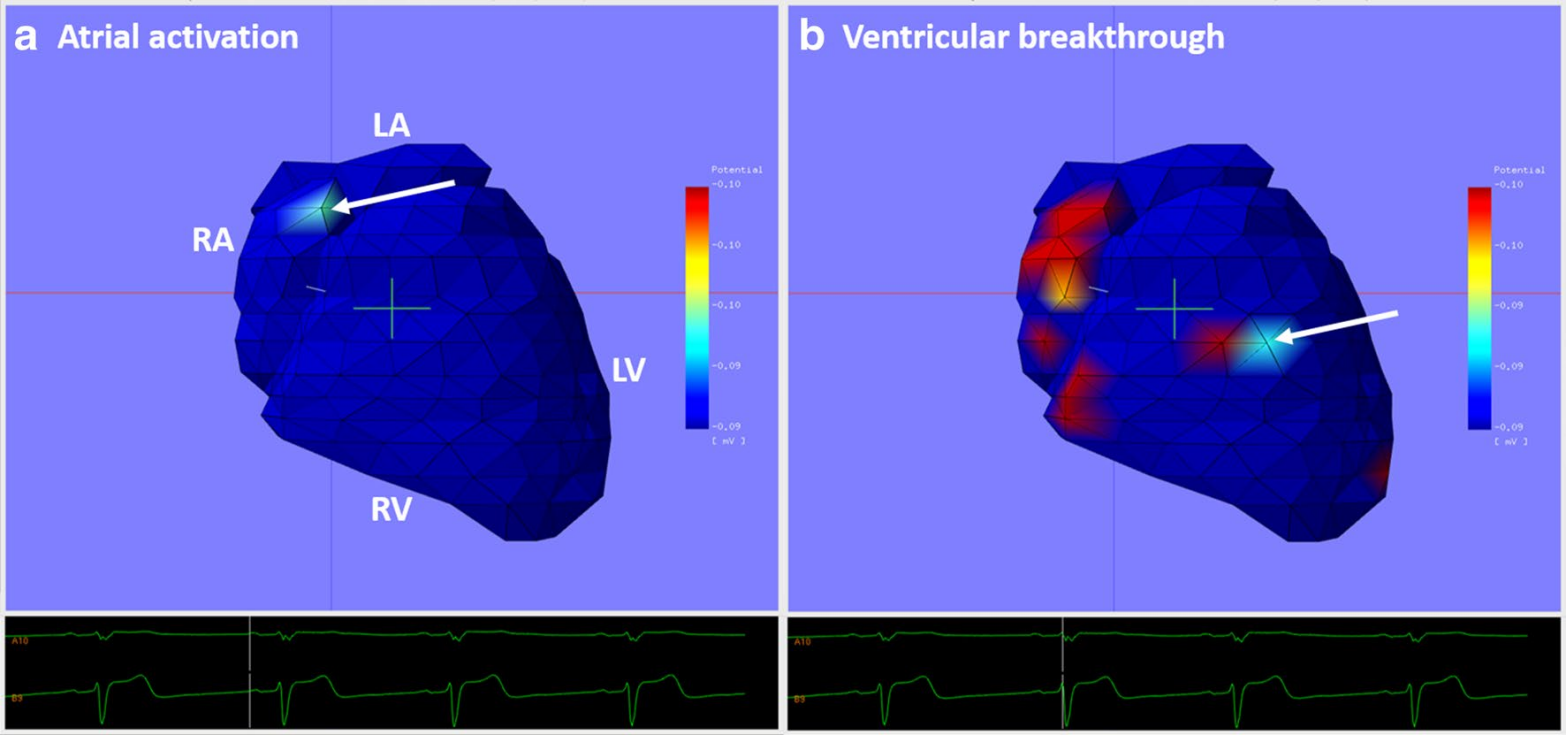
IPM in a healthy volunteer during sinus rhythm. The left panel **a** depicts the IPM at the start of the atrial depolarization (P wave) and the right panel **b** depicts the IPM at the start of the ventricular depolarization (QRS). The first point of atrial activation is observed near the entrance of the Superior Vena Cave (*white arrow*) into the Right Atrium. The first point of ventricular epicardial breakthrough is observed at the RV free wall (*white arrow*) at the site of the moderator band

During sinus rhythm, the first point of activation on the potential map of all study patients was located near the crista terminalis of the superior vena cave into the right atrium (Fig. 2a).

The first point of ventricular epicardial breakthrough was located at the right ventricular free wall. This corresponds to the location where the moderator band was attached to the ventricular myocardium (Fig. 2b). During this time, a high potential distribution is observed at the right atrial wall, indicating the atrial repolarization (Fig. 2a).

### Simulations

**Fig. 3:**
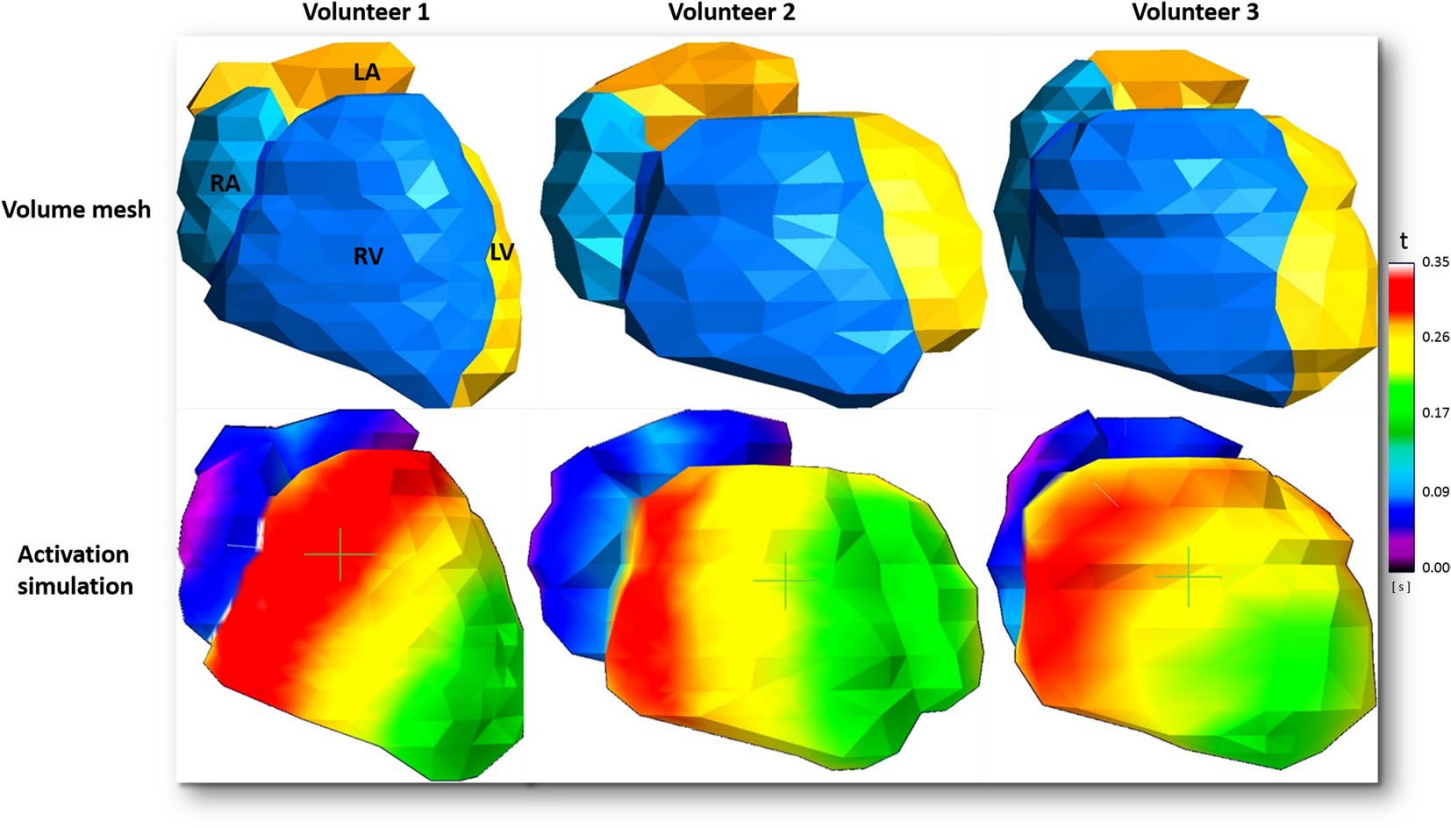
Simulation of normal cardiac activation on whole-heart computational meshes. *Top row* depicts computational volume meshes generated from the three different segmentations. *Bottom row* depicts isochrone maps generated after performing a cardiac activation simulation on the computational mesh

The cardiac activation cycle (sinus rhythm) was successfully simulated on all healthy computational meshes. The generated virtual isochrones maps depicted cardiac activation patterns in accordance with the literature (Fig. 3; Additional file 2: Video 2) [14].

**Fig. 4:**
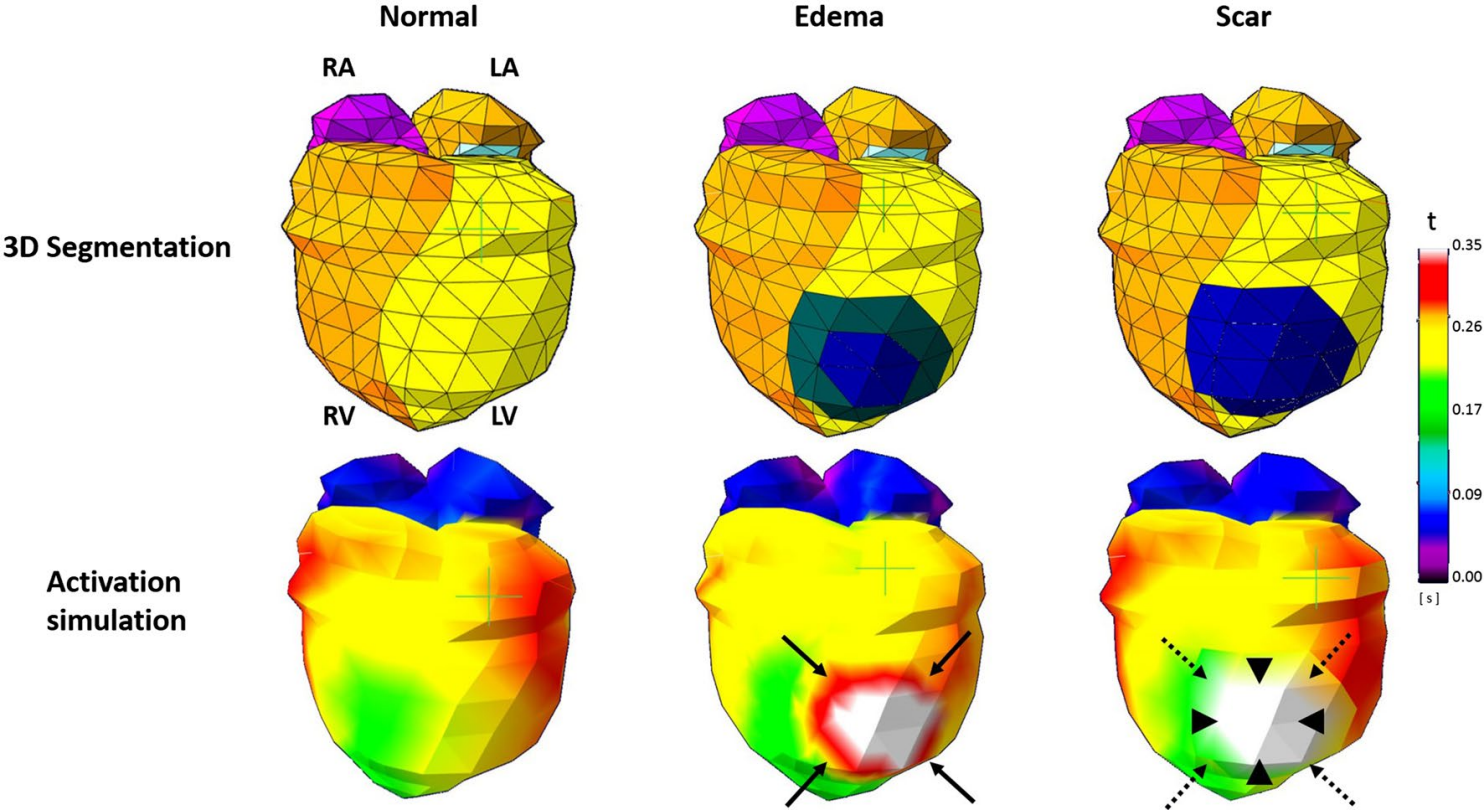
Feasibility of cardiac activation simulation on customized meshes with pre-defined structural abnormalities. *Top row* depicts the segmentations created using the custom made tool. The *middle* and *right column* segmentations have a pre-defined structural abnormality. The impact of these abnormalities is visible on the corresponding simulations. A delayed activation can be observed in the isochrone map for regions with edema (*solid black arrows*). The computational mesh containing a transmural scar (*black arrowheads* and *dotted black arrows*), demonstrates a total conduction block

The simulations for computational meshes with structural abnormalities (edema and scar), were also completed successfully. The mesh with a pre-defined virtual edema region demonstrated a delayed activation pattern (Fig. 4), whereas, a complete conductional block was observed for the computational mesh containing virtual transmural scar (Fig. 4).

## Discussion

To our knowledge, this is the first study to integrate and evaluate IPM and cardiac activation simulations within a clinically applicable whole-heart workflow. The proposed computational workflow provided accurate IPM reconstructions and enabled patient-specific simulations to be performed.

The use of MRI enables visualization and incorporation of tissue-characteristics in computational meshes. In addition, an integrated IPM and simulation based approach facilitates a comprehensive assessment of arrhythmias and underlying substrate. These two factors significantly contribute towards the clinical applicability of this workflow and offer a unique environment for the development and evaluation of patient tailored therapeutic strategies.

### IPM

The IPM localized the origin of atrial activity to the anatomically known location of the sinus node [15], suggesting the correctness of the model. These results imply that sinus node function and also dysfunction may be non-invasively characterized and assessed using IPM.

The ability to reconstruct atrial depolarization and repolarization can also contribute to novel clinical insights in the electrical substrate of complex supra-ventricular tachycardias such as left atrial flutter and atrial fibrillation.

### Simulations

The simulations of sinus rhythm performed on the computational meshes generated reliable results when compared to measurements reported in the literature [14]. Simulations performed on the personalized meshes incorporating virtual edema and scar resulted in a different activation pattern with delayed conduction and conduction block respectively.

These observations illustrate the wide range of simulations which can be obtained utilizing this simulation model. This can be clinically relevant for patients presenting with arrhythmias with a history of a disease associated with fibrosis such as myocardial infarction and myocarditis.

### Whole-heart computational model

Although only a few pathologies involve the atria and ventricles simultaneously, a combined assessment remains relevant for a comprehensive study of atrio-ventricular electro-mechanical coupling and electrical interaction.

The atrial contraction presents such an example. It has been reported that pressure modulation due to atrial contractions can remotely alter the electrical behavior and activation pattern of the ventricular myocytes [16, 17]. A whole-heart model is required to incorporate such complex relations and to provide physiologically accurate simulations.

## Limitations

The simulations performed in this study used a standardized set of epicardial potentials recorded in a structurally normal human heart. Furthermore, the currently used simulation algorithm applied fixed conduction velocities for the cardiac volumes.

However, the aim of the current study was to develop a clinically applicable and reliable method for isochrones generation. The evaluation of this algorithm could be successfully performed using one set (atrial and ventricular) of epicardial potentials. The generated simulation results were accurate for all subjects when compared to previous descriptions in the literature [14]. Therefore, the absence of patient specific epicardial potentials and usage of a fixed conduction model was not considered as a limitation.

The customized meshes were constructed to test the feasibility of incorporating tissue properties. The conductivity and speed values for edema and scar tissue were based on estimates. Although, this can be considered as a substantial limitation, the simulations with patient-specific geometries (incorporating scar and edema) demonstrated realistic results, and underline the feasibility of the simulation algorithm.

A next step would be combining simulations with reconstructed IPM in order to non-invasively characterize tissue.

### Future directions

Future research will focus on evaluating the IPM algorithm for patients undergoing catheter ablation for ectopic ventricular beats. The simulation algorithm will be further evaluated and optimized in patients with structural heart disease and arrhythmias. Furthermore, future work will perform comparison between simulations, reconstructed IPM and invasively acquired epicardial potentials to evaluate potential reconstruction errors related to patient specific differences in epicardial potentials.

## Conclusion

The proposed finite element model based whole-heart computational workflow offers an integrated platform for cardiac electrical assessment using IPM and simulations. The IPM algorithm can accurately reconstruct reliable cardiac activation sequences from BSP’s. The simulation model was able to incorporate patient-specific electrical parameters and rapidly perform whole-heart cardiac activation simulations. The use of MRI substantially contributes towards the clinical applicability.

This workflow offers the prospect to improve patient selection and personalize therapeutic strategies for interventional electrophysiological procedures. Future studies should investigate the role of this innovative approach for analysis of complex atrial and ventricular arrhythmias.

## Additional files

10.1186/s12967-016-0902-0 Dynamic overview of the ventricular topological elements constructed in the custom made tool.

10.1186/s12967-016-0902-0 Rotational view of the simulated cardiac isochrones.
